# A genomic analysis of disease-resistance genes encoding nucleotide binding sites in *Sorghum bicolor*

**DOI:** 10.1590/S1415-47572010005000036

**Published:** 2010-06-01

**Authors:** Xiao Cheng, Haiyang Jiang, Yang Zhao, Yexiong Qian, Suwen Zhu, Beijiu Cheng

**Affiliations:** School of Life Science, Anhui Agricultural University, Hefei, AnhuiChina

**Keywords:** bioinformatics, disease resistance gene, nucleotide binding site, *Sorghum bicolor*

## Abstract

A large set of candidate nucleotide-binding site (NBS)-encoding genes related to disease resistance *was* identified in the sorghum (*Sorghum bicolor*) genome. These resistance (R) genes were characterized based on their structural diversity, physical chromosomal location and phylogenetic relationships. Based on their N-terminal motifs and leucine-rich repeats (LRR), 50 non-regular NBS genes and 224 regular NBS genes were identified in 274 candidate NBS genes. The regular NBS genes were classified into ten types: CNL, CN, CNLX, CNX, CNXL, CXN, NX, N, NL and NLX. The vast majority (97%) of NBS genes occurred in gene clusters, indicating extensive gene duplication in the evolution of *S. bicolor* NBS genes. Analysis of the *S. bicolor* NBS phylogenetic tree revealed two major clades. Most NBS genes were located at the distal tip of the long arms of the ten sorghum chromosomes, a pattern significantly different from rice and *Arabidopsis*, the NBS genes of which have a random chromosomal distribution.

## Introduction

Most higher plants are susceptible to infection by a variety of pathogens. A common mechanism of defense against pathogens involves a “hypersensitive response” that results in apoptosis or natural cell death of infected cells, a process in which resistance or R genes play an important role (Dhalowal and Uchimiya, 1999). Resistance genes encode proteins involved in plant resistance to a variety of pathogens, including bacteria, viruses, fungi, oomycetes, nematodes and insects. The best characterized R genes encode products that contain a nucleotide-binding site (NBS) and a series of leucine-rich repeats (LRR) (Lupas *et al.*, 1991). The NBS sequences have been extensively used to identify and classify plant R genes based on their content of conserved motifs. The NBS-LRR genes are the most common type of R genes that have been sequenced and disease resistance is the only function that has been ascribed to them. Meyers *et al.* (1999) divided the NBS-LRR genes into two sub-types based on the N-terminal structure of the encoded proteins, *i.e.*, those with an N-terminal region homologous to the Toll/interleukin-1 receptor (TIR) and those with no such homologous region (so-called non-TIR NBS-LRR or non-TNL genes). Non-TNL genes are of two types: those with a coiled-coil (CC) domain in the N-terminal region (that is shorter than in CNL genes), referred to as CC-NBS-LRR, and those with an unknown structure (X) in the N-terminus (XNL), referred to as X-NBS-LRR.

According to the specific gene-for-gene hypothesis proposed by Flor (1971), interaction between the host plant and its pathogens involves avirulence genes that encode a product recognized by a specific R gene of the host plant; virulence thus represents a loss-of-function in the pathogen and a failure to trigger the defense response that in turn facilitates the occurrence of disease. If an R gene recognizes the avr gene product of the pathogen then a signal transduction cascade that leads to differential gene expression and disease resistance is activated. A proper understanding of the host plant-pathogen relationship and determination of the structure, function and mechanism of R genes are important areas of research in phytopathology and plant disease resistance (Baker *et al.*, 1997).

The genome sequence of *Sorghum bicolor* has been reported (Paterson *et al.*, 2009). In this work, we used this database to investigate the diversity of *S. bicolor* R genes and assess their importance in the mechanisms of disease resistance. In the future, the cloning of R genes (The *Arabidopsis* Genome Initiative 2000; Venter *et al.*, 2001; Goff *et al.*, 2002; Yu *et al.*, 2005) should help to accelerate the breeding of disease-resistant *S. bicolor*.

## Methods

###  The *Sorghum bicolor* genome

The complete genome sequence of *S. bicolor* was downloaded from the phytozome database.

###  Identification of NBS-encoding genes

By using keywords such as “NBS” and “resistance” to search 117 gene groups in GenBank we identified 143 genes with a cereal NBS motif. This NBS domain nucleotide sequence was then used as a query in BLASTN searches for possible homologs encoded in the *S. bicolor* genome. The threshold expectation value was set to 10^-3^, a value determined empirically to filter out most of the spurious hits. This step was crucial in identifying the maximum number of candidate genes. The nucleotide sequences of candidate NBS genes were used as queries to find homologs in the *S. bicolor* genome by BLAST searches (p value = 0.001). All of the sequences that met the requirements were analyzed by using the Pfam (*P*rotein *family*) database in order to remove genes that did not contain NBS gene sequences. Each of the candidate sequences was checked manually by using available annotations in GenBank to confirm that they encoded the corresponding NBS candidate proteins. The sequences were then aligned with ClustalW using MEGA 3.1 software (Kumar *et al.*, 2004) and identical sequences located in longer sequences or genes were eliminated.

###  Classification of NBS genes

The NBS amino acid sequences derived from the standard NBS gene region of the Pfam database by using the hidden Markov model (HMM), starting from a pre-P-Loop structure to the end of the MHDV motif, generally contained 260-300 amino acids. (The four amino acid sequence MHDV is a key feature of most NBS-LRR proteins; Meyers *et al.*, 2003). The N-terminus precedes the pre-P-Loop structure and the LRR region follows the MHDV motif. All of the corresponding NBS candidate proteins were surveyed to determine whether they encoded TIR, CC, NBS or LRR motifs. This survey was based on the Pfam database (http://pfam.janelia.org) and used SMART (*S*imple *M*odular *A*rchitecture *R*esearch *T*ool) protein motif analysis (Schultz *et al.*, 1998) and COILS, a program to detect coiled coil (CC) domains (Lupas *et al.*, 1991) and used with a threshold = 0.9).

###  Physical location of NBS genes on sorghum chromosomes

The software DNATOOLS was used to construct local databases with the complete *S. bicolor* nucleotide sequence and the starting positions of all NBS genes on each chromosome were obtained using tBLASTn (p value = .001). This method was used to confirm the physical locations of all NBS genes. Finally, a chromosomal map showing the physical location of all regular NBS resistance genes was generated with Genome Pixelizer software.

###  Sequence alignment and phylogenetic analysis

Multiple alignments of amino acid sequences were done with the NBS region based on the Pfam results. Phylogenetic trees were constructed using MEGA 3.1 based on the bootstrap neighbor-joining (NJ) method (bootstrap = 1000) with a Kimura 2-parameter model; these trees were subsequently used to analyze the evolutionary relationships among sorghum NBS disease-resistant genes.

## Results

###  Identification and classification of NBS genes

Two hundred and eighty-three prospective R genes were identified in the *S. bicolor* genome, nine of which were subsequently found not to be NBS-encoding genes. Analysis of the remaining 274 genes with an NBS structure (based on the Pfam database) identified 224 genes with highly conserved NBS regions and a complete open reading frame (ORF). Based on sequence differences in the N-terminal and LRR regions, these 224 NBS disease-resistance genes were classified into six types: NBS, NBS-LRR, NBS-X, X-NBS, NBS-LRR-X and NBS-X-LRR (the N-terminal region of most regular NBS genes contained unknown motifs represented as X). Fifty genes had highly differentiated NBS regions with structures that varied considerably from the other 224 genes. The N-terminal regions of these genes were also short (less than 2/3 the length of the normal NBS proteins). These genes were therefore classified as non-regular NBS genes. All non-redundant candidate NBS genes were also surveyed using Pfam, SMART and COILS to determine whether they encoded TIR, CC, NBS or LRR motifs (see Materials and Methods). This information on protein motifs and domains was used to classify the NBS-encoding genes into subgroups. Finally, 143 genes containing a CC motif were identified and divided into subtypes (COILS-NBS, COILS-NBS-LRR, COILS-NBS-LRR-X, COILS-NBS-X); these genes accounted for 63.8% of all standard NBS genes (Table 1).

At least 30,434, 45,555, 27,000, 37,544 and 36,338 protein-coding genes have been identified in the fully sequenced grape (Hulbert *et al.*, 2001; Jaillon *et al.*, 2001), poplar (Tuskan *et al.*, 2006), *Arabidopsis* (Richly *et al.*, 2002), rice (Zhou *et al.*, 2004) and *Sorghum* genomes, respectively. NBS-LRR genes accounted for approximately 1.51%, 0.72%, 0.53%, 1.23% and 0.18% of all predicted ORFs in these five species, respectively. Although the absolute number of NBS-LRR genes in *Sorghum* was similar to rice, the relative proportion of these genes was significantly lower than in the vine grape, poplar, *Arabidopsis* and rice genomes (Table 1).

###  Chromosomal locations of *Sorghum* NBS genes

**Figure 1:**
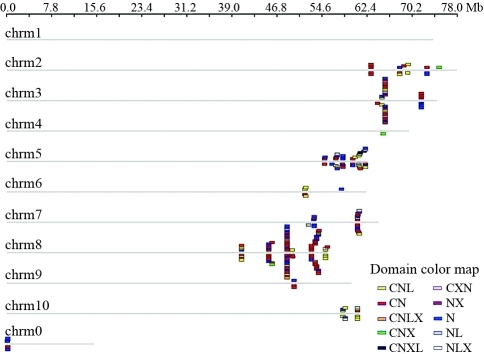
Physical locations of the *Sorghum bicolor* NBS *R* genes. The boxes above and below the chromosomes (chrm; gray bars) indicate the approximate locations of the ten categories of NBS genes indicated in Table 1. Unmapped genes are shown on Chrm0.

Standard NBS type disease-resistance genes were identified on the ten *Sorghum* chromosomes by using Genome Pixelizer software and classified into one of ten categories (CNL, CN, CNLX, CNX, CNXL, CXN, NX, N, NL and NLX), each represented by a different color (Figure 1).

Based on the location of individual NBS genes, 268 of the 274 *Sorghum* NBS-encoding genes were mapped on the ten chromosomes. The remaining genes were located on a sequence not yet linked to a chromosome (referred to as chromosome 0 in Figure 1). Of the 268 anchored NBS genes, 223 (83.2%) were located on chromosomes 3, 5, 7 and 8 (41, 45, 24 and 113 NBS-encoding genes on each respective chromosome); chromosome 1 had no NBS-encoding gene. No CC type genes were identified, while the chromosomal distribution of non-CC type genes showed an obvious pattern. As defined by Holub (2001), a gene cluster is a region in which two neighboring homologous genes are < 200 kb apart. Of the genes analyzed here, 217 were located in 20 gene clusters, each with an average of 12 genes (twice the average number of genes/cluster in rice). The largest cluster contained 36 NBS genes on chromosome 8. Most *Sorghum* NBS genes fell into multi-gene clusters, a distribution similar with that of rice and *Arabidopsis.* However, in contrast to the latter two species, most of the sorghum NBS gene clusters were located at the distal end of each chromosome (Figure 1).

###  Phylogenetic analysis of regular NBS genes in *Sorghum*

**Figure 2:**
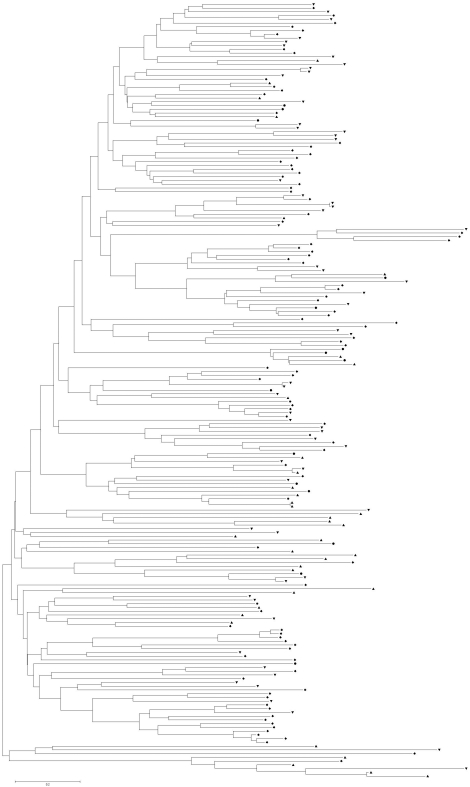
Phylogenetic analysis of the NBS genes in the *Sorghum bicolor* genome based on the amino acid sequences in the NBS domain. ? CN, q N, p CNL and • NL. CN indicates Coiled-coil NBS R-genes, N indicates NBS R-genes, CNL indicates Coiled-coil NBS-LRR R-genes and NL indicates NBS-LRR R-genes. The scale bar represents 20 nucleotide substitutions per 100 nucleotides.

The amino acid sequences of the NBS region contained highly conserved motifs with high homology that allowed sequence alignment and the construction of phylogenetic trees to assess the relationships among the standard NBS disease-resistance genes in the sorghum genome (Figure 2). Two hundred and twenty-four standard NBS alleles were selected for sequence alignment and phylogenetic tree construction. The other 50 non-regular NBS genes were excluded because of their short N-termini.

The phylogenetic tree of the NBS domain in *Arabidopsis* (a dicotyledon) has two distinct clades, whereas there is no clear division of NBS regions in rice (a monocotyledon), which has a divergent, star-shaped distribution. Figure 2 shows that the phylogenetic tree of the *Sorghum* NBS domain also contained two major clades, which suggests that the evolutionary pattern of the *Sorghum* NBS domain was similar to that of *Arabidopsis*. Phylogenetic analysis revealed no independent clade for the NBS-LRR gene with the CC-motif, which suggested a complex pattern of evolution (Figure 2).

## Discussion

By using the NBS genes of other plants as queries we were able to identify 224 *Sorghum* genes with highly conserved NBS regions. No TIR-encoding genes were found among these 224 genes, a finding similar to that reported for rice (only one TIR-X gene identified) (Zhou *et al.*, 2004) and maize. In contrast, the number of TIR encoding genes varies from 98 in *Arabidopsis* to 78 in poplar. TIR and non-TIR sequences are remarkably different among dicots and monocots, which suggest an ancient origin and subsequent divergence between the two NBS gene types; TIR-encoding genes appear to have been lost in grass genomes. The deduced NBS-LRR proteins were divided into two subfamilies, TIR-NBS-LRR (TNL) and non-TNL, based on their N-terminal features. Whereas dicotyledons such as *Arabidopsis* and poplar (Yang *et al.*, 2008) have many NBS resistance genes that encode proteins with N-termini containing a TIR structure, no such structures were found among the *Sorghum* NBS-LRR genes; this situation is similar to that of other monocotyledons such as rice and maize. It is unclear why grass genomes have a greatly reduced set of TIR-encoding genes, although gene loss or the lack of TIR gene amplification are possible explanations. Phylogenetic analysis of the NBS genes revealed differences between *Sorghum* and other plants. Thus, unlike rice which shows no major clades in its NBS domain (Zhou *et al.*, 2004), two major clades were identified in the *Sorghum* sequences. This difference may reflect different forms of duplication in the past, although the actual mechansim remains unclear.

There was considerable variation in the chromosomal distribution of NBS genes in *Sorghum*. For example, there were no NBS genes on chromosome 1, whereas chromosome 8 contained 90 NBS genes; this situation was similar to that of rice in which 25% of the R-like genes were present on chromosome 11. This finding indicates that there are chromosomal hot spots in which the NBS genes share greater homology; these genes may have originated by tandem duplication and subsequently diverged under selective pressure. Vine grape and poplar contain 77 and 75 clusters, respectively, including 445 and 281 NBS genes with an average of 5.78 and 3.75 NBS members per cluster. In *Sorghum*, 97% of the NBS genes were located in gene clusters, a much higher proportion than in vine grape and poplar; there was also potentially more duplication and more multi-gene families in the *Sorghum* genome compared to the latter two species. The *Sorghum* NBS resistance genes were distributed in clusters in the distal part of the chromosomes. This peculiar location may reflect the long period of contact between *Sorghum* and its pathogen, with the disease-resistance genes expanding by duplication and rearrangement to eventually form distributional hot spots on the chromosomes. Phylogenetic analysis of the *Sorghum* NBS genes revealed clusters containing NBS genes from the same or different families. Mixed clusters could arise by chromosomal rearrangement and transposition or by genomic duplication and mutations. Unequal crossing over based on regions of homology could also expand the cluster sizes.

## Figures and Tables

**Table 1:** The number of genes that encode domains similar to NBS genes in the *Sorghum bicolor* genome.

Predicted protein domains	Letter code	Number of genes
Regular NBS genes		
CC-NBS-LRR	CNL	36
CC-NBS	CN	99
CC-NBS-LRR-X	CNLX	2
CC-NBS-X	CNX	4
CC-NBS-X-LRR	CNXL	1
CC-X-NBS	CXN	1
NBS-X	NX	3
NBS	N	61
NBS- LRR	NL	16
NBS-LRR-X	NLX	1
Total regular NBS genes		224
Non-regular NBS genes		
NBS	-	40
NBS-LRR	-	10
Total non-regular genes		50
Total NBS genes		274
